# Systematic comparison of post-column isotope dilution using LC-CO-IRMS with qNMR for amino acid purity determination

**DOI:** 10.1007/s00216-019-02116-2

**Published:** 2019-09-12

**Authors:** Philip J. H. Dunn, Dmitry Malinovsky, Eli Achtar, Cailean Clarkson, Heidi Goenaga-Infante

**Affiliations:** National Measurement Laboratory, LGC Limited, Queen’s Road, Teddington, Middlesex, TW11 0LY UK

**Keywords:** Post-column isotope dilution, Purity, Isotope ratio mass spectrometry, Measurement uncertainty, Quantitative NMR

## Abstract

Determination of the purity of a substance traceable to the International System of Units (SI) is important for the production of reference materials affording traceability in quantitative measurements. Post-column isotope dilution using liquid chromatography-chemical oxidation-isotope ratio mass spectrometry (ID-LC-CO-IRMS) has previously been suggested as a means to determine the purity of organic compounds; however, the lack of an uncertainty budget has prevented assessment of the utility this approach until now. In this work, the previously published ID-LC-CO-IRMS methods have not only been improved by direct gravimetric determination of the mass flow of ^13^C-labelled spike but also a comprehensive uncertainty budget has been established. This enabled direct comparison of the well-characterised ID-LC-CO-IRMS method to quantitative nuclear magnetic resonance spectroscopy (qNMR) for purity determination using valine as the model compound. The ID-LC-CO-IRMS and qNMR methods provided results that were in agreement within the associated measurement uncertainty for the purity of a sample of valine of (97.1 ± 4.7)% and (99.64 ± 0.20)%, respectively (expanded uncertainties, *k* = 2). The magnitude of the measurement uncertainty for ID-LC-CO-IRMS determination of valine purity precludes the use of this method for determination of purity by direct analysis of the main component in the majority of situations; however, a mass balance approach is expected to result in significantly improved measurement uncertainty.

**Electronic supplementary material:**

The online version of this article (10.1007/s00216-019-02116-2) contains supplementary material, which is available to authorized users.

## Introduction

Many methods for assigning purity values such as high-performance liquid chromatography with ultra violet/visible detection (HPLC-UV/Vis) or gas chromatography with flame ionisation detection (GC-FID) that target the impurities of a calibrant often suffer from non-uniformity of response between analytes. In turn, this leads to large uncertainties associated with impurities that cannot be calibrated adequately due to a lack of reference materials of those impurities. The paucity of reference materials for impurity compounds is also a problem for isotope dilution mass spectrometry (IDMS)–based methods where isotopically labelled analogues (with ^2^H or ^13^C substitutions) for each of the impurities are required for species-specific IDMS quantification which again must be sourced or synthesised and then characterised. This burden of impurity identification followed by sourcing and assignment of purity values on those impurities is the largest contributor to the time and cost of developing a reference material of a pure substance [1]. Therefore, there is a real need for further development of capabilities for direct assay methodologies for chemical purity of organic calibrants.

Some previous attempts to find a ‘universal detector’ that allows direct correlation of signal intensity to mass fraction (e.g. charged aerosol detection [2–4] and evaporative light-scattering detection [5, 6]) have failed to find a robust, sensitive and generally applicable solution. Quantitative nuclear magnetic resonance spectroscopy (qNMR) has emerged as one of the few methodologies capable of providing direct assay purity determinations which are traceable to the International System of Units (SI) [7–9]. It is, however, desirable to have a second, independent technique for method validation and to provide confirmatory measurements during reference material characterisation.

Post-column IDMS (also known as species-unspecific IDMS) has been demonstrated to provide accurate quantitative results [10–12]. In 2009, the concept of using the combustion interface that links gas chromatographs to isotope ratio mass spectrometry (IRMS) instruments (GC-C-IRMS) to allow the use of a single spike compound for post-column IDMS analysis of a range of organic compounds was presented [13]. There had been earlier work using GC-C-IRMS instrumentation for IDMS analyses but using the species-specific IDMS approach [14–16]. The post-column IDMS was further developed [17, 18] and extended to a similar approach using the chemical oxidation interface linking liquid chromatography to IRMS instrumentation (LC-CO-IRMS) [19].

These post-column IDMS approaches used an internal standard (IS) of known purity to determine the mass flow of the isotopically labelled spike [17–19]; however, this presents a number of challenges for both LC- and GC-based applications. Firstly, the IS must be chromatographically resolved from all other components within the analyte solution. This is particularly important for LC separations as the chemical oxidation precludes the use of carbon-containing mobile phases limiting the separation chemistry that can be applied. Wide peaks with substantial isotopic fractionation across the peak width of the order of hundreds of permil are typical in LC-CO-IRMS analyses making baseline resolution of compounds vital [20]. Secondly, the IS must exhibit similar conversion characteristics within the combustion or chemical oxidation interface to the compounds of interest. Combustion interfaces for GC coupled to IRMS are relatively free from such bias although the presence of some elements such as silicon within analytes can cause problems resulting from the deposition of non-volatile species within the reactor [20], while others such as fluorine can cause the production of reactive species that have been reported to cause damage to instrument components downstream [21]. Persulfate oxidation employed by commercial LC-CO-IRMS systems exhibits difficulty in oxidising carbon atoms bonded to two or three nitrogen atoms or within aromatic N-heterocycles to carbon dioxide [19]. Similarly, halogenated compounds can also be difficult to oxidise when three halogens are bound to the same carbon atom such as trichloroacetic acid and trifluoroacetic acid [22]. A suite of IS compounds of known purity may therefore be necessary. Finally, not all IS compounds are suitable for use with all chromatographies, again requiring a range of known purity materials.

Post-column IDMS can be applied without the need for an IS of known purity—provided that the mass flow of the spike can be determined independently. For LC separations, this can be achieved via gravimetric determination of spike mass flow using a balance [23]. While post-column IDMS with HPLC-ICP-MS has been successfully employed for mass fraction determination of elemental species, it has not yet been applied for quantification of organic compounds (e.g. those without an elemental tag visible to ICP-MS). Furthermore, the few publications describing post-column IDMS of organic compounds using IRMS instrumentation have reported standard deviation of replicate analyses rather than a full measurement uncertainty budget (e.g. [13, 17–19]). Without a comprehensive estimation of measurement uncertainty, it is difficult to assess the method in terms of usefulness for purity determination.

In this work, we have attempted to address the shortcomings of previous methods through the quantification of valine using LC-CO-IRMS with post-column IDMS (referred to herein as ID-LC-CO-IRMS). The method has been improved by removing the need for an internal standard of known purity for spike flow determination. Also, a full uncertainty budget is provided for this approach for the first time. The improved ID-LC-CO-IRMS methodology is here compared to qNMR analyses of the same valine material, not simply in terms of mass fraction and measurement uncertainty but also with regard to other considerations such as cost and turnaround time that may be important to measurement stakeholders (people and organisations with an interest in the measurement results including the analyst themselves, individuals/managers within the same organisation, external, potentially fee-paying individuals/companies/bodies including the police/courts/regulators/research councils, amongst others). Some suggestions for future research and improvements to the reported ID-LC-CO-IRMS method are also discussed.

## Materials and methods

### Notation

All uncertainties reported in this manuscript are expanded uncertainties with 95% confidence. The *k*-factor required to obtain this confidence level has been specified in each instance. A value of *k* = 2 was used where degrees of freedom were sufficiently large. In some cases, larger values of *k* were required to achieve the same level of confidence and these were obtained from Student’s t-distribution.

### ID-LC-CO-IRMS

Analyses were performed using an Ultimate 3000 series autosampler and four channel pump coupled via an LC IsoLink chemical oxidation interface to a Delta V isotope ratio mass spectrometer (all Thermo Scientific, Bremen, Germany). A SIELC Primesep A column (3.2 × 250 mm, 5-μm particle size, 100 Å pore size; SiELC Technologies Ltd., Prospect Heights, IL, USA) was used to separate the valine. The mobile phase was 100% 18.2 MΩ cm^−1^ water (Elga Ltd., High Wycombe, UK) for 10 min, then a gradient to 100% 0.024 M H_2_SO_4_ (Sigma-Aldrich, Poole, UK) over the next 30 min with the sulphuric acid used until the end of the run. Each analysis included a 30-min equilibration time at the end where the mobile phase was returned to 18.2 MΩ cm^−1^ water. The flow rate of mobile phase was 300 μL min^−1^ throughout.

The LC IsoLink was slightly modified from the standard configuration. Instead of two separate reservoirs of oxidation reagents for 1.5 M phosphoric acid and 0.1 g mL^−1^ sodium persulfate (both Sigma-Aldrich, Poole, UK), in 18.2 MΩ cm^−1^ water each pumped by a separate piston pump, the oxidation reagents were combined into a single solution of equivalent concentration in each reagent and pumped using the LC IsoLink “acid” pump at 40 μL min^−1^. The “ox” pump of the LC IsoLink was used to add the spike solution of ^13^C-labelled D-glucose (Sigma-Aldrich, Poole, UK) in 18.2 MΩ cm^−1^ water at a concentration of 100–120 μg g^−1^ and a flow rate of 20 μL min^−1^. This allowed complete equilibration between the CO_2_ derived from the sample and from the spike within the chemical oxidation reactor. The reservoir of spike was placed on to a balance (GA200D, Ohaus, PineBrook, NJ, USA) and the mass recorded regularly while the solution was being pumped into the instrumentation to allow determination of the mass flow of the spike.

The mass spectrometer was fitted with electronically switchable amplifiers on the middle collector allowing two different configurations. The first had the usual 100× amplification of *m/z* 45 relative to *m/z* 44 while the second had both of these collectors at equal amplification. The latter was used during analyses involving highly ^13^C-enriched materials (e.g. spike characterisation and IDMS analyses). Proprietary software (Isodat version 3.0) was used for data acquisition and the data exported to Microsoft Excel for further calculations. Peak areas were recorded in pA s^−1^ rather than the default mV s^−1^ to ensure that the amplification of the *m/z* 45 signal could be correctly accounted for. The data reduction process is described in detail in subsequent sections.

### FIA-CO-IRMS—natural abundance measurements

FIA-CO-IRMS analysis was performed using the same instrumentation as above with the following differences: (i) there was no HPLC column between sample injection and chemical oxidation stages resulting in a bulk carbon isotope ratio measurement and (ii) the “ox” pump was not used; i.e. only mixed sodium persulfate and phosphoric acid reagents were added to the mobile phase prior to the chemical oxidation reactor using the ‘acid’ pump.

The sample valine was analysed by FIA-CO-IRMS against synthetic isotope mixtures of glycine previously prepared for absolute carbon isotope ratio measurements. The *R*_*glycine*_(^13^C/^12^C) values of the synthetic isotope mixtures were (0.010543 ± 0.000024), (0.011070 ± 0.000025) and (0.011546 ± 0.000026) (expanded uncertainties, *k* = 2 [24]). Raw instrumental data were the mean and standard deviation of the integrated ion current ratios for the working gas (*n* = 6 per analysis) and sample gas (*n* = 5 per analysis). Each material was therefore analysed in a separate run. Data analysis was performed in Microsoft Excel using a Kragten-type [25] template to (i) calculate raw isotope ratios (*r*(^13^C/^12^C) values) for the sample gas peaks using the data reduction scheme previously described [26]; (ii) perform a blank correction using a mass balance approach where the blank isotopic composition and signal amplitude were determined from the replicate analysis of procedural blanks at the beginning and end of each analytical sequence; and (iii) normalisation of the measured isotope delta values to the SI-traceable absolute isotope ratio scale using the known and measured values of the SIMs.

### FIA-CO-IRMS—^13^C-enriched measurements

For calibration of the ^13^C-labelled spike, the same FIA-CO-IRMS system was used but with equal amplification on both Faraday collectors 2 and 3 of the mass spectrometer (i.e. those measuring at *m/z* 44 and 45). Determination of the spike isotopic composition was achieved by single point calibration using previously characterised ^13^C-labelled glycine with *R*(^13^C/^12^C) = (211 ± 4) (expanded uncertainty, *k* = 2 [24]). The data analysis was again performed in a Kragten-type spreadsheet and included raw *R*(^13^C/^12^C) estimation from the recorded *R*(^45^(CO_2_)/^44^(CO_2_)) values using the SSH ^17^O algorithm approach [27], but using updated values for the absolute isotope ratios of VPDB and *λ* (we have described this data analysis approach in more detail elsewhere [28]). No working gas was used for these analyses as both the sample (i.e. ^13^C-labelled D-glucose) and reference material (i.e. ^13^C-labelled glycine) could be injected within the same analytical run.

### NMR structural confirmation

Approximately 10 mg each of the analyte and internal standard maleic acid (Sigma-Aldrich) was dissolved in deuterium oxide (D_2_O) for valine or dimethylsulfoxide-d_6_ (DMSO-d_6_) for the spike, vortexed, sonicated and transferred to a 5-mm NMR tube for analysis. To enable structural confirmation of the ^13^C-labelled D-glucose spike and natural abundance valine, a ^1^H and ^13^C (spike only) NMR spectrum and correlation spectroscopy (COSY), heteronuclear single quantum coherence (HSQC), and heteronuclear multi bond correlation (HMBC) spectra were obtained using a Bruker Avance 600 MHz NMR spectrometer with a 5-mm broadband inverse probe. For the ^1^H NMR analyses, the following parameters were used: 16 scans, receiver delay of 1 s, temperature of 298.0 K and spectral width of 20.0 ppm.

### qNMR quantitative analysis

Approximately 20 mg of the spike or 13 mg of the valine together with 10 mg of a maleic acid internal standard (Sigma-Aldrich, previously calibrated in-house against NIST 350b Benzoic Acid) was accurately weighed and dissolved in 1 mL of D_2_O (valine) or DMSO-d_6_ (spike). The solution was vortex mixed and sonicated for 1 min then transferred to a 5-mm NMR tube for analysis.

Three independent solutions were prepared and qNMR experiments were acquired in triplicate for each solution. Due to the additional spectrum complexity arising from the ^13^C coupling of the ^13^C-labelled glucose, only the α and β anomeric ^1^H signals could be used for quantitation. These two analyte signals did not appear to overlap with any impurity signals and were therefore considered representative of the ^13^C_6_ D-glucose molecule. For the valine, the analyte signal at 3.74 ppm was considered representative of the molecule. ^1^H qNMR analyses were performed on a Bruker Avance 600 MHz NMR using the parameters listed in Table 1.

**Table 1:** Instrumental parameters for the purity determination of the spike ^13^C_6_ D-glucose and of the valine by qNMR

Parameter	Value	
	Spike	Valine
Number of scans	16	32
Relaxation delay	60 s	
Spectral width	20.0 ppm	
Temperature	298.0 K	
FID processing software	Topspin 3.5 pl 2	Topspin 3.5 pl 2
Baseline correction	Manual, polynomial baseline correction	
Signal integration	Manual	Manual, excluding ^13^C satellites
Analyte signal(s)	4.90 and 4.27 ppm	3.74 ppm
Maleic acid signal	6.28 ppm	
SINO valve, analyte signal	827	20,874
SINO valve, standard signal	15,817	99,744

The purity of the analytes was determined using the following equation:

1 $$ {x}_a=\frac{I_a}{I_{std}}\frac{n_{std}(H)}{n_a(H)}\frac{M_{r,a}}{M_{r, std}}\frac{m_{std}}{m_a}{x}_{std} $$

where the subscripts *a* and *std* refer to the analyte and maleic acid internal standard, respectively; *I* is the integrated peak area of the quantification signal; *n*(H) is the number of hydrogen atoms; *M*_*r*_ is the molecular weight; *m* is the weighted mass; and *x* is the mass fraction. The uncertainty in the obtained purity was obtained from Eq. (3) based on the approach of Saed Al-Deen [29]:

2 $$ u\left({x}_a\right)=\sqrt{{\left(\frac{s\left({x}_a\right)}{x_a}\right)}^2\times {\left(\frac{u\left({M}_{r,a}\right)}{M_{r,a}}\right)}^2\times {\left(\frac{u\left({M}_{r, std}\right)}{M_{r, std}}\right)}^2\times {\left(\frac{u\left({m}_a\right)}{m_a}\right)}^2\times {\left(\frac{u\left({m}_{std}\right)}{m_{std}}\right)}^2\times {\left(\frac{s\left({x}_{std}\right)}{x_{std}}\right)}^2} $$

where the subscripts *s* and *std* refer to the D-glucose sample and maleic acid internal standard, respectively; s(*x*) is the standard deviation of replicate analyses of *x*; and *u* is the standard uncertainty.

## Results and discussion

The post-column IDMS equation is shown in Eq. (3).

3 $$ {MF}_s={MF}_{sp}\times \frac{A_{r,s}(C)}{A_{r, sp}(C)}\times \frac{x_{sp}\left({}^{13}C\right)}{x_s\left({}^{12}C\right)}\times \left(\frac{R_{blend}\left({}^{12}C{/}^{13}C\right)-{R}_{sp}\left({}^{12}C{/}^{13}C\right)}{1-{R}_{blend}\left({}^{12}C{/}^{13}C\right)\times {R}_s\left({}^{13}C{/}^{12}C\right)}\right) $$

where the subscripts *s* and *sp* refer to the sample and spike, respectively, while *blend* refers to the mixture of sample and spike; *MF* is the mass flow of carbon; *A*_*r*_(C) is the atomic weight of carbon; *x*(^13^C) and *x*(^12^C) are the amount fractions of these isotopes; and *R*(^12^C/^13^C) and *R*(^13^C/^12^C) are absolute isotope ratios for the appropriate material. Equation (1) can be applied in two different ways: the first involves integrating the signals for *m/z* = 44 and 45 prior to application of the post-column IDMS equation (referred to herein as ‘peakwise’), while the second applies the equation to each data point to produce a mass flow chromatogram which is then integrated (referred to herein as ‘pointwise’). Both the peakwise and pointwise approaches require the determination of each of the terms which are discussed in the subsequent sections.

### Determination of *MF*_*sp*_

Previous application of ID-LC-CO-IRMS used ^13^C-labelled sodium bicarbonate as a spike [19]. This is a material which is quantitatively converted to CO_2_ by the chemical oxidation within commercial LC-CO-IRMS interfaces, thereby making it ideal for applications where an internal standard compound of known purity is used to estimate spike mass flow. In this work, the spike mass flow was determined gravimetrically, rather than from an internal or external standard and therefore, it was necessary for the spike itself to be characterised for purity. Sodium bicarbonate is unsuitable for purity analysis by qNMR as it only has one proton and therefore ^13^C-labelled D-glucose was chosen as an alternative as its purity could be assessed by qNMR while it is still quantitatively converted to CO_2_ within the oxidation reactor.

The mass fraction of the ^13^C-labelled D-glucose within the spike material (i.e. the purity) was determined to be (98.42 ± 1.01)% (expanded uncertainty, *k* = 2.45) by qNMR. This result is traceable to the SI via the kilogramme realised by use of ISO 17025 calibrated balances and an internal in-house standard certified for purity by mass fraction (itself traceable to NIST SRM 350b benzoic acid).

The spike solution was prepared gravimetrically in 18.2 MΩ cm^−1^ water, accounting for the purity determined above at a typical mass concentration of 100–120 μg g^−1^ with a relative expanded uncertainty (*k* = 2) of < 1% (e.g. (118.66 ± 0.52) μg g^−1^). The spike solution was placed on to a balance (GA200D, Ohaus, PineBrook, NJ, USA) and the mass recorded regularly while the solution was being pumped into the instrumentation, beginning before and ending after each analytical sequence. The calibration of the balance was checked prior to use using SI-traceable check weights. The spike mass flow was taken to be the gradient of the linear regression between mass lost against time elapsed. The uncertainty associated with the spike mass flow was taken to be the standard error of the gradient. Each ID-LC-CO-IRMS analytical run required 3 h due to the chromatographic conditions amenable to LC-CO-IRMS separation of amino acids and each analytical sequence included multiple runs of sample and blanks and therefore, the spike mass flow was typically recorded over a period of > 72 h. The flow rate obtained was approximately 300 μg s^−1^ (e.g. (305.85 ± 0.53) μg s^−1^). The square of the Pearson product moment correlation coefficient through the data points was > 0.999.

The stability of the spike flow rate was also confirmed by examination of the *m/z* = 44 and 45 background signals while the spike was being pumped through the system prior to each analytical sequence. Without the presence of the spike, the standard deviation of the signals obtained over a 300-s window for *m/z* 44 monitored on a Faraday collector with 3 × 10^8^ amplification was typically < 1 mV. With the spike, this increased slightly but was still below the 10 mV threshold suggested by the manufacturer. When equal amplification was used for both Faraday collectors monitoring *m/z* 44 and 45, the standard deviations of both signals were comparable and also below 10 mV.

### Determination of *A*_*r,s*_(C) and *A*_*r,sp*_(C)

The atomic weight of carbon within the sample and spike can be determined from the isotope amount fractions *x*(^12^C) and *x*(^13^C) and the atomic weights of the isotopes of carbon, *A*_*r*_(^12^C) and *A*_*r*_(^13^C), using Eqs. (4) and (5):

4 $$ {A}_{r,s}(C)=\left({x}_s\Big({}^{12}C\times {A}_r\left({}^{12}C\right)\right)+\left({x}_s\left({}^{13}C\right)\times {A}_r\left({}^{13}C\right)\right) $$

5 $$ {A}_{r, sp}(C)=\left({x}_{sp}\left({}^{12}C\right)\times {A}_r\left({}^{12}C\right)\right)+\left({x}_{sp}\left({}^{13}C\right)\times {A}_r\left({}^{13}C\right)\right) $$

### Determination of *x*_*s*_(^12^C) and *x*_*sp*_(^13^C)

The isotope amount fractions of carbon in the sample and spike can be determined from their absolute isotope ratios *R*_*s*_(^13^C/^12^C) and *R*_*sp*_(^13^C/^12^C), respectively, using Eqs. (6) to (9).

6 $$ {x}_s\left({}^{12}C\right)=\left(\frac{1}{1+{R}_s\left({}^{13}C{/}^{12}C\right)}\right) $$

7 $$ {x}_s\left({}^{13}C\right)=1-{x}_s\left({}^{12}C\right) $$

8 $$ {x}_{sp}\left({}^{13}C\right)=\left(\frac{R_{sp}\left({}^{13}C{/}^{12}C\right)}{1+{R}_{sp}\left({}^{13}C{/}^{12}C\right)}\right) $$

9 $$ {x}_{sp}\left({}^{12}C\right)=1-{x}_{sp}\left({}^{13}C\right) $$

### Determination of *R*_*sp*_(^12^C/^13^C)

**Fig. 1:**
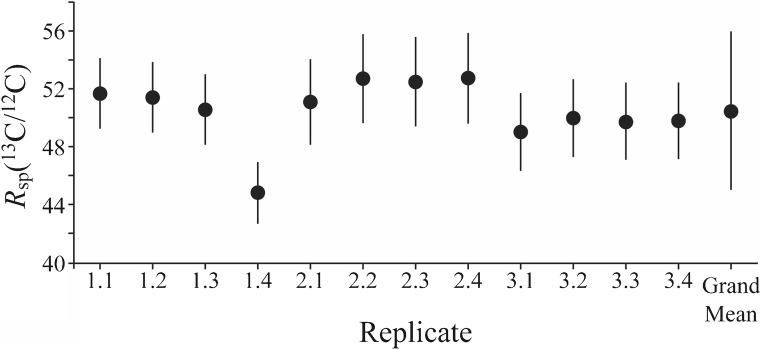
*R*_*sp*_(^13^C/^12^C) values obtained for the ^13^C-labelled D-glucose by single point calibration against a previously characterised, ^13^C-enriched glycine. Three subsamples of the ^13^C-labelled D-glucose were analysed and each analysis included four repeat injections

The *R*_*sp*_(^12^C/^13^C) value was determined as the reciprocal of the *R*_*sp*_(^13^C/^12^C) value which could be measured by offline FIA-CO-IRMS analysis using previously characterised ^13^C-labelled glycine with *R*_*glycine*_(^13^C/^12^C) = (211 ± 4) (expanded uncertainty, *k* = 2) for one point calibration [24]. Each analytical run consisted of alternate injections of the two ^13^C-labelled compounds (five injections of the glycine and four of the spike). Three separate spike calibration analysis runs were carried out and the grand mean of the *R*_*sp*_(^13^C/^12^C) values obtained was (50.5 ± 5.5) (expanded uncertainty, *k* = 2, Fig. 1). This expanded uncertainty includes contributions from the assigned isotopic composition of the ^13^C-labelled glycine calibrant together with the standard deviation of instrumentally measured parameters obtained from the replicate analyses. Although one point calibration is not ideal, the paucity of materials calibrated for carbon isotope ratio that are highly enriched in ^13^C affords little alternative. The contribution of the *R*_*sp*_(^13^C/^12^C) value to the uncertainty budget for purity determination is very low and therefore the use of a non-ideal approach can be justified.

### Determination of *R*_*s*_(^13^C/^12^C)

The *R*_*s*_(^13^C/^12^C) value was determined by offline FIA-CO-IRMS analysis using various synthetic isotope mixtures of glycine previously characterised for absolute carbon isotope ratio for normalisation of results [22]. In this way, the use of the standard isotope delta equation and the absolute isotope ratio of the virtual zero point VPDB were not needed [28]. The absolute isotope ratio of the valine obtained was *R*_Valine_(^13^C/^12^C) = (0.010675 ± 0.000573) (expanded uncertainty, *k* = 2) based upon the analysis of three independently prepared solutions of valine. The uncertainty budget showed that the measured ion current ratios for the sample and working gas peaks from analysis of the valine solutions and of the SIMs were the dominant sources of uncertainty.

### Determination of *R*_*blend*_(^12^C/^13^C)

#### Need for ^17^O correction

During carbon isotope ratio analysis of natural organic compounds converted to carbon dioxide or of commercial CO_2_, the ^12^C^17^O^16^O isotopologue contributes approximately between 6 and 7% to the *m/z* 45 signal which is itself approximately 1% of the amplitude of the *m/z* 44 signal. There are a number of so-called ^17^O algorithms which have been formulated to overcome this [27, 30, 31]. In ^13^C-labelled compounds (i.e. non-natural isotopic abundance of carbon), the oxygen isotopic composition may also be non-natural. It can therefore be expected that the ^17^O abundance of the spike D-glucose may also be significantly different to natural abundance and therefore may contribute to a greater, or indeed lesser, extent to the *m/z* 45 signal.

While the oxygen isotopic composition of the spike could be measured and used to determine an appropriate correction for the presence of ^17^O in the CO_2_ resulting from chemical oxidation of the spike, a simpler option is to ensure that the oxygen isotopic composition of all peaks in chromatograms is identical. In other words, the aim “is to try to ‘reproduce’ the same oxygen isotopic composition in the different blends measured so that it can compensate for the effects of the oxygen contribution [32].” This approach has previously been applied during the analysis and certification of a steroid reference material and for exact-matching ID-IRMS analysis of ethanol [33, 34]. It is also the basis for the analysis of carbonate reference materials by EA-IRMS whereby the roasting of carbonate within the combustion reactor ensures that the resultant CO_2_ has equivalent oxygen isotopic composition to the combustion of organic materials [35].

The majority of the oxygen in sample CO_2_ peaks will be derived from the oxidation reagents which should have a uniform isotopic composition within a single batch of reagent solution. Provided that the equivalence of oxygen isotopic composition can be assured for the blends measured, then neglecting to perform a ^17^O correction would be acceptable. To test this assumption, preliminary experimental data were analysed in two different ways: firstly including a correction for ^17^O following the usual SSH algorithm [27] and secondly simply using the raw peak area ratio of the *m/z* 45 and 44 traces. There was no difference in the sample mass flow calculated within uncertainty between these two approaches and therefore, it was deemed that a correction for ^17^O need not be applied during future data handling; hence, the *R*(^12^C/^13^C) and *R*(^13^C/^12^C) values were assumed to be equivalent to the instrumentally measured *R*(^44^(CO_2_)/^45^(CO_2_)) and *R*(^45^(CO_2_)/^44^(CO_2_)) values, respectively.

#### Test for complete conversion of valine to CO_2_

During determination of *R*_*s*_(^13^C/^12^C) for the valine (above), the peak area of the valine without the spike present in the FIA-CO-IRMS system could be evaluated. During ID-LC-CO-IRMS analyses, the peak area of the valine *m/z* 44 signal resulting from injection of the same volume of the same valine solution while the spike was introduced into the system was the same within uncertainty. This demonstrated that the efficiency of the chemical oxidation of the valine to carbon dioxide was not reduced by the constant presence of the spike within the reactor.

#### Percentage carbon in spike and analyte

The percentage carbon content in spike and analyte compounds was determined using the standard atomic weights for H, N and O together with the atomic weights of carbon determined above. The ^13^C-labelled glucose spike was therefore 41.9% carbon while the natural abundance valine was 51.3% carbon. The percentage carbon content was used to convert between mass flows of carbon and mass flows of the corresponding compound.

#### Determination of injection mass

One aim of this work was to avoid the interconversion of volumes and masses during calculation stages and therefore, it was necessary to determine the mass of injected solution, rather than simply the injection volume. This would be unnecessary in cases where an internal or external standard of known purity was used to determine spike mass flow provided that both sample and standard were injected in equal volumes using the same syringe. Sample vials were weighed before and after each analytical sequence. The total mass of solution injected was then divided by the number of injections from that vial to give the typical mass of solution per injection. Repeated injections from the same vial can damage the septum within the vial cap leading to the potential for loss of solution by evaporation and therefore, no more than four injections from the same vial were performed during analytical sequences. All injections used the same volume which was set within the software and the same syringe.

The mass of a 10-μL injection of the valine solution in 18.2 MΩ cm^−1^ water was usually slightly larger than 10 mg (e.g. (10.2838 ± 0.0003) mg). The mass concentration of valine in the injected solution was determined gravimetrically during preparation. Typically, this was between 60 and 80 μg g^−1^ with a relative expanded uncertainty of < 4%.

#### Integration and correction for contributions of blank and background

The instrumental software (Thermo Scientific Isodat v 3.0) was used to integrate the ion current signals from the Faraday collectors. The integration parameters can be found in Table 2. This allowed consistent integration of the Gaussian sample gas peaks in the chromatograms; however, the need to account accurately for the spike signal necessitated additional calculation steps. The *m/z* 44 signal consisted of the integrated Gaussian peak plus the background signal which was determined using the peak width and background value recorded by the instrumental software (‘BGD 44’ in Isodat). There was typically no peak in the *m/z* 45 signal as the spike was in great excess to the isotopologues of CO_2_ with *m/z* 45 arising from the sample—the area recorded was simply a result of fluctuations in the signal over the time period of the peak. The peak area for *m/z* 45 was therefore assumed to be the background value recorded by Isodat (‘BGD 45’) multiplied by the peak width.

**Table 2:** Integration settings applied/selected in Isodat

Parameter	Value
Start slope	3.33 pA s^−1^
End slope	6.66 pA s^−1^
Background type	Individual BGD
History	5 s
Time shift	y (limit 1 data point)
Peak detection	*m/z* 44

**Fig. 2:**
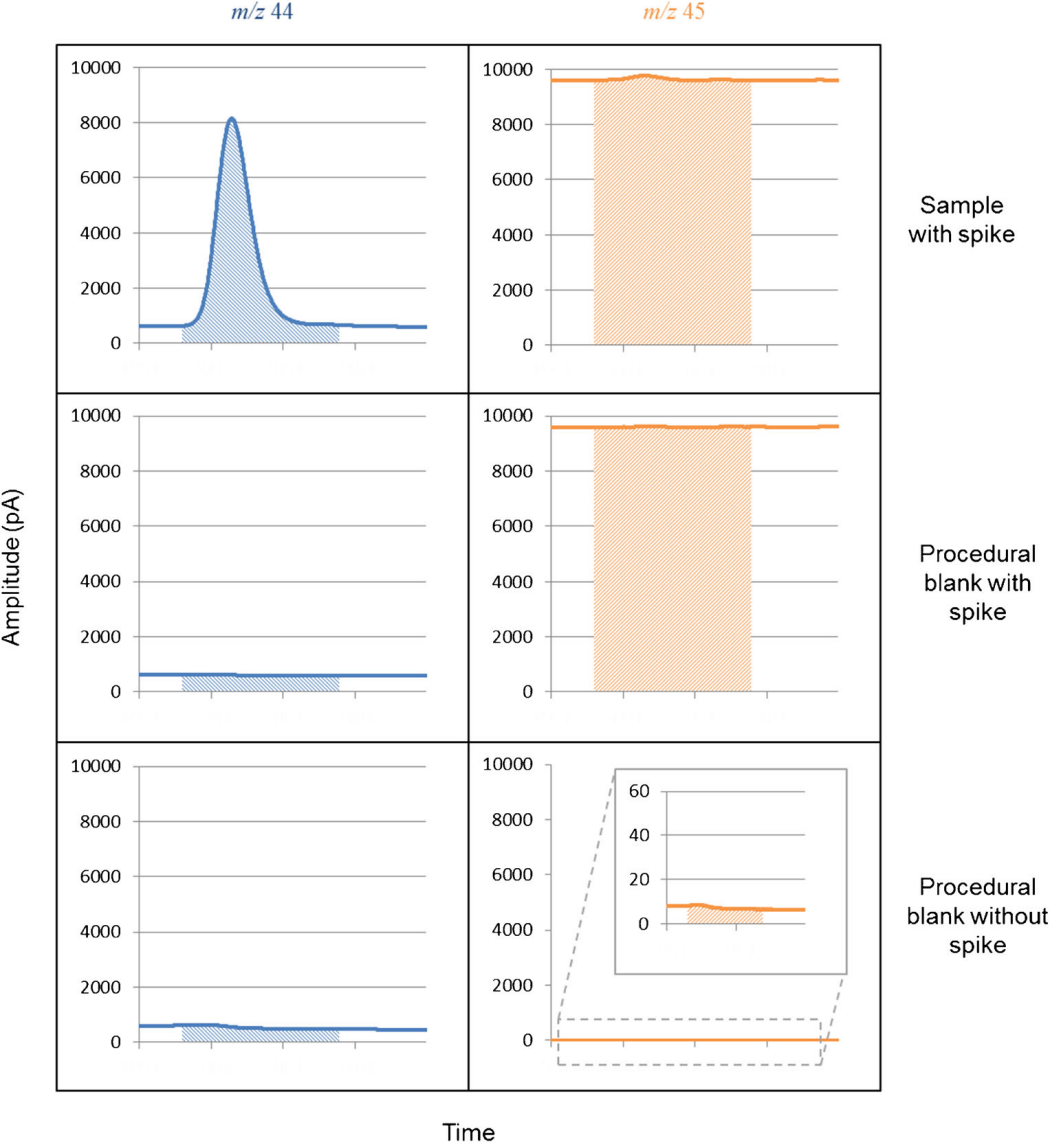
Partial chromatograms for the *m/z* 44 and 45 signals highlighting the peak areas for ID-LC-CO-IRMS analysis using the spike for the valine and procedural blank as well as for the blank without the spike. All chromatograms are shown to the same scale for easy comparison

Procedural blanks were prepared using the same batch of 18.2 MΩ cm^−1^ water as for the preparation of the sample solutions. These were analysed before and after each sample solution and examined for the presence of peaks coinciding with the analyte(s) of interest. There was no evidence that the procedural blanks contained any valine; however, the signals for *m/z* 44 and 45 during the time period where valine was expected to elute were not zero but ~ 600 pA and ~ 9600 pA, respectively. This background signal was visible to the same extent during valine analysis when the spike was present (again *m/z* 44 ~ 600 pA and *m/z* 45 ~ 9600 pA). Analysis of the same valine and procedural blank solutions with no spike present but using the same amplifier configuration also yielded a background carbon signal, but of reduced magnitude of ~ 400 pA for *m/z* 44 and 5 pA for *m/z* 45. This demonstrated that the spike itself contributed ~ 200 pA to the *m/z* 44 and ~ 9595 pA to the *m/z* 45 signals. The 45/44 ratio of these spike signals is 48, which is very similar to the *R*_*sp*_(^13^C/^12^C) value of (50.5 ± 5.5) noted earlier lending credence to the background values. The remaining background signal during the IDMS analyses not due to the spike (i.e. the ~ 400 pA for *m/z* 44 and 5 pA for *m/z* 45) is likely due to a combination of carbon sources including column bleed, dissolved carbon dioxide or other carbon-containing molecules within the mobile phase, the oxidation reagents, the spike solution or the water used to dissolve samples, and any other source of carbon within the instrumentation. The various background levels and the integrated peak areas described are illustrated schematically in Fig. 2.

The contribution of the non-spike background carbon signal must be accounted for with a background correction. This correction should result in zero mass flow for the sample when there isno sample peak (i.e. for the analysis of a procedural blank). This background correction was applied by calculating the appropriate areas for *m/z* 44 and 45 with no sample gas peak (i.e. the areas depicted in the middle panes of Fig. 2), using these to calculate the background ion current ratio, which could then be introduced into Eq. (1) to determine the mass flow of background carbon which was then subtracted from the mass flow of sample carbon. While this may seem as though, the areas depicted in the middle panes of Fig. 2 are therefore included initially, only to be removed later; this is not the case as the peak areas are introduced as ratios into Eq. (1). The initial inclusion of the background areas is vital if the peak area of the spike signal at *m/z* = 45 is to be correctly determined—without the background area to this signal the integrated peak area for *m/z* = 45 would be the same whether or not spike was present in the system. This correction does assume that the level of column bleed or other background carbon is the same for procedural blanks as for sample analyses; however, this is not unreasonable.

### Calculation of *MF*_*s*_: peakwise approach

The *R*_*blend*_(^12^C/^13^C) value calculated using the background corrected peak areas described above was input into Eq. (3) together with the other terms to result in the mass flow of the sample carbon, which could be converted into a mass flow of the sample using the known elemental composition of the valine. The uncertainty in the *MF*_*s*_ value was estimated by carrying out the data reduction stages within a Kragten spreadsheet [25] and an example is provided within the Electronic Supplementary Material (ESM). In this way, the uncertainty associated with each term within Eq. (3) was determined either from instrumental measurements or from values obtained in the literature as described in the preceding sections and then combined into the measurement uncertainty for a particular result.

### Calculation of *MF*_*s*_: pointwise approach

The individual data points for the *m/z* 44 and 45 signals were exported to Excel. For each time point, the post-column IDMS equation was applied using the values of *MF*_*SP*_, *A*_*r,s*_(C), *A*_*r,sp*_(C), *x*_*sp*_(C), *x*_*s*_(C), *R*_*sp*_(^12^C/^13^C) and *R*_*s*_(^13^C/^12^C) as determined above together with the instantaneous *R*_*blend*_(^12^C/^13^C) equivalent to the *m/z* 44 signal divided by the *m/z* 45 signal. This resulted in the calculated *MF*_*s*_ for each time point which could be plotted as a mass flow chromatogram. To ensure that the background was accounted for, the *MF*_*s*_ values were corrected by subtraction of the mean of the five *MF*_*s*_ values immediately preceding the integration interval (this is an analogous method to the background correction applied by the Isodat Individual BGD algorithm, Table 2). Integration of these corrected *MF*_*s*_ values would thereby result in zero mass flow if there were no peak present which would not otherwise be the case. In this way, it is again possible to correct for the presence of background carbon from column bleed etc. Integration was carried out using both a trapezoidal method and Simpson’s rule [21], although these approaches gave identical answers.

The uncertainty in the mass flow determined via the pointwise approach is somewhat complicated to estimate. Each of the terms within Eq. (1) other than *R*_*blend*_(^12^C/^13^C) has a standard uncertainty that could be combined using simple rules. The equation is then applied to each data point in the ratio chromatogram and the resultant mass flow chromatogram is integrated. Application of the IDMS equation adds the same degree of uncertainty to each data point and therefore, the same relative uncertainty in the integrated peak area should be expected as for each data point. However, as each data point is highly correlated to the adjacent points and that accounting for this correlation is difficult, the uncertainty in the integrated area is more difficult to establish. Moreover, integration generally results in an underestimate of the true area of the peak which impacts the associated uncertainty. Perhaps due to the complexity involved, previously reported post-column IDMS results that have applied the pointwise approach have simply indicated the standard deviation of replicate analyses rather than a complete measurement uncertainty budget [12, 13, 17–19].

### Comparison of peakwise and pointwise approaches

The ESM where the peakwise and pointwise approaches are applied to the same raw data from a single IDMS run. The peakwise approach assumes that the areas and widths of the peaks within the *m/z* 44 and 45 signals are exact and results in a mass of sample injected of (577.7 ± 5.4) ng (expanded uncertainty with *k* = 2). The pointwise approach yielded 574.7 ng regardless of integration approach applied; however, measurement uncertainty cannot be reliably established from only a single analysis. These results agree within the expanded uncertainty of the peakwise result and therefore, it does not matter what the uncertainty associated with the pointwise result is—within uncertainty both approaches yield identical results.

While previous ID-LC-CO-IRMS analyses have applied a pointwise approach for application of Eq. (1), there are a number of reasons why a peakwise approach as described above is better. Firstly, as noted above, if Eq. (1) is applied to each pair of data points from *m/z* 44 and 45, it becomes more difficult to propagate the uncertainty from application of IDMS equation to the final sample mass obtained. Secondly, if the IDMS equation is applied using integrated peak areas, then the integration package available within the instrumental software can be applied, while if a mass flow chromatogram must be constructed prior to integration, then the use of the proprietary software becomes more difficult. Integration can be performed offline from the instrument within a variety of software packages including spreadsheets; however, some of the integration parameters that are easily accessed within proprietary may be significantly more difficult to vary (or to apply consistently) offline. Thirdly, the peakwise approach uses ratios within Eq. (1) that have all been obtained in the same way (i.e. using instrumental software and before any calculations) which is not the case for the pointwise approach. We therefore selected the peakwise approach for subsequent analyses.

### Measurement uncertainty for purity determination by ID-LC-CO-IRMS

**Fig. 3:**
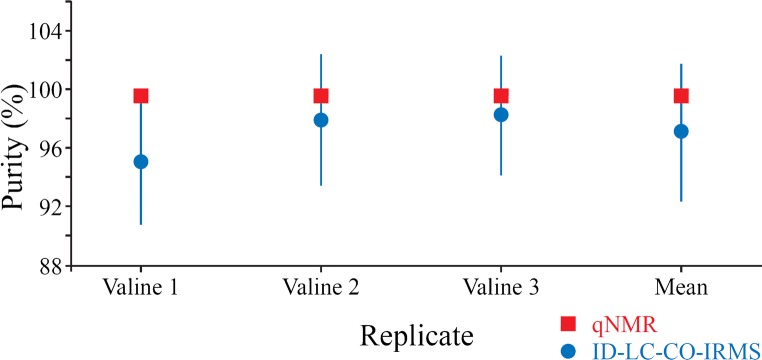
Purity of the valine determined by ID-LC-CO-IRMS compared to the result obtained by qNMR. Error bars show the expanded uncertainty with *k* = 2 which have not been truncated for the ID-LC-CO-IRMS results (error bars for the qNMR results are 0.20% and therefore smaller than the markers in the plot)

To determine the purity of the valine, the mass injected determined by ID-LC-CO-IRMS analysis was compared to the result obtained from gravimetric preparation of the solutions (during which a 100% purity of the valine had been assumed) and expressed as a percentage: The valine sample was independently prepared in solution for analysis three times at a range of concentration of 60 to 80 μg g^−1^. Two subsamples of each solution were analysed several times each for a combined total of seven ID-LC-CO-IRMS measurements for each of the three valine solutions. These were combined to give a single purity value for each solution with associated uncertainty. In addition, a mean value from all measurements of the valine (*n* = 21) was also calculated which was (97.1 ± 4.7%) (Fig. 3); however, this includes values above 100% purity and therefore the upper limit should be truncated [36]. The 95% confidence interval for the purity of valine as determined by ID-LC-CO-IRMS was therefore from 92.4% to 100%. Expressed as relative expanded uncertainty, this interval becomes from 92.3% to 100%.

The measurement uncertainties shown in Fig. 3 for the mean purity of the valine obtained comprised the contributions listed in Table 3. The largest contributions were from the measured terms contributing to the *R*_*blend*_(^12^C/^13^C) value as well as the gravimetrically determined concentration of the valine solution.

**Table 3:** Measurement uncertainty budget for determination of the purity of valine by ID-LC-CO-IRMS

Parameter	Contribution to uncertainty (%)
Repeatability	18.6
Valine concentration	39.7
*R*_*sp*_(^13^C/^12^C)	0.2
Spike concentration	3.2
Spike flow rate	0.5
*R*_*blend*_(^12^C/^13^C)	37.8
Other components	< 0.1

While the measurement uncertainty determined here is larger than previously reported indications of precision (e.g. [13, 17–19]) this is not altogether surprising given that the repeatability contribution to uncertainty is less than 20%. Furthermore, the previous precision estimates were based upon the use of an internal standard to quantify the spike mass flow, rather than the gravimetric approach employed in this work.

### General imitations of post-column IDMS

Applications of IDMS for quantitation require that the sample and spike are equilibrated as early as possible within the measurement process to ensure that loss of the analyte can be compensated for. When post-column IDMS is employed, the sample is equilibrated with the spike only after initial handling (e.g. dissolution), injection into the chromatograph and chromatographic separation of analyte from other components present. Loss of analyte can occur at potentially each of these stages which would give rise to biased results. Similarly, equilibration between sample and spike must be complete and may be more difficult to achieve for post-column IDMS than for offline IDMS.

In this work, the likelihood of incomplete equilibration between sample- and spike-derived carbon dioxide was deemed to be minimal: In the modified LC-CO-IRMS instrumentation, the oxidation reagents, spike and sample all pass through the oxidation reactor contemporaneously. The reactor itself consists of a long, tightly coiled capillary surrounding the heating element. The resulting carbon dioxide is removed from the mobile phase within a gas separation unit using a partially permeable membrane and a counter-flow of helium. The carbon dioxide then passes through two sequential gas drying membranes (Nafion®) before entering the open split and being transported to the ion source of the mass spectrometer. There is therefore ample opportunity for equilibration between CO_2_ derived from the sample and that derived from the spike before entering the mass spectrometer.

Potential loss of valine between sampling from the vial using the autosampler syringe and the addition of the spike within the instrumentation is certainly possible. To investigate this, the qNMR-derived purity of the valine was included within the calculation of the concentration of the three valine solutions. In this way, the difference between the gravimetrically known amount of valine within an injection and the amount obtained by post-column IDMS provides an indication of the recovery of the valine (rather than its purity). For the three solutions valine shown in Fig. 3, these recoveries were (95.4 ± 4.2%), (98.3 ± 4.2%) and (98.6 ± 4.2%), respectively (expanded uncertainties, *k* = 2). The overall recovery for valine was (97.5 ± 4.7%) (expanded uncertainty, *k* = 2) which encompasses 100% within the uncertainty bounds. Note that this recovery only accounts for the difference in mass of valine between that in the volume of solution lost from the HPLC vial during injection, and that detected/calculated by post-column IDMS. It is possible that some of the valine solution removed from the vial remains within the syringe or adheres to the syringe needle and is not injected into the mobile phase stream; however, this might be detectable in blank injections that immediately follow valine injections. Such blanks did not show any sign of a peak corresponding to valine.

A further check for complete transfer of the valine through the instrumentation is to compare the carbon isotope delta for valine separated using the same chromatography as employed for post-column IDMS, but without the addition of the spike, to the same isotope delta obtained by offline bulk analysis. If there were to be a significant difference in these two isotope delta values, then either the minor impurities within the valine have highly anomalous isotope delta values, or there has been fractionation of the valine during chromatographic separation. The carbon isotope delta value for the valine was determined offline by FIA-CO-IRMS using four reference materials for normalisation to the Vienna PeeDee Belemnite (VPDB) scale. These reference materials were AE672a glycine with *δ*_VPDB_(^13^C/^12^C) = (− 42.12 ± 0.42) ‰ (expanded uncertainty, *k* = 2); USGS40 L-glutamic acid with *δ*_VPDB_(^13^C/^12^C) = (− 26.39 ± 0.08) ‰ (expanded uncertainty, *k* = 2); IAEA-CH-6 sucrose with *δ*_VPDB_(^13^C/^12^C) = (− 10.45 ± 0.06) ‰ (expanded uncertainty, *k* = 2); and USGS41 L-glutamic acid with *δ*_VPDB_(^13^C/^12^C) = (+ 37.63 ± 0.10) ‰ (expanded uncertainty, *k* = 2). The result obtained for the valine from four measurements of two independent solutions was *δ*_VPDB_(^13^C/^12^C) = (−11.06 ± 0.75) ‰ (expanded uncertainty, *k* = 4.3). The equivalent isotope delta for the valine obtained by LC-CO-IRMS from one measurement of each of six independently prepared solutions using two reference materials for normalisation (USGS40 and USGS41 L-glutamic acids) was *δ*_VPDB_(^13^C/^12^C) = (− 10.72 ± 0.17) ‰ (expanded uncertainty, *k* = 2.6). These two isotope deltas overlap within their expanded uncertainties so there is no detectable fractionation of valine resulting from passing through the LC-CO-IRMS instrumentation.

### Comparison of ID-LC-CO-IRMS with qNMR

The same valine sample was also analysed by qNMR to determine its purity resulting in a value of (99.64 ± 0.20) % (expanded uncertainty, *k* = 1.96). Within the expanded uncertainty, the values obtained for the purity of valine by qNMR overlap with those obtained by ID-LC-CO-IRMS. However, the expanded measurement uncertainty for qNMR was 0.2% in terms of purity for the valine while the value obtained by ID-LC-CO-IRMS was significantly higher (4.7%). That the measurement uncertainty for the latter was larger was not surprising. As no internal standard of known purity was used to determine the spike mass flow (for reasons discussed in the introduction), the determination of the spike mass flow was carried out during the course of the sample measurement. This approach therefore does not benefit from a large number of repeated measurements of the same property as would be the case during characterisation of an internal standard of known purity.

The ID-LC-CO-IRMS results were also consistently lower than those for qNMR. This potential bias could either result from over estimation of the concentration of valine in the solutions during gravimetric preparation or from underestimation of the amount of valine in the solution during quantification. For the latter, there are several different factors which could result in a low-biased purity value, which fall under the general limitations of post-column IDMS discussed above. These include incomplete oxidation of valine to carbon dioxide, incomplete equilibrium between sample and spike CO_2_ prior to the source of the mass spectrometer and loss of valine within the instrumentation prior to spike addition. There is also the possibility of underestimation of peak area: As noted earlier, integration of a chromatographic peak results in an underestimate of the true area, but this bias will be small. The concentrations of the three solutions valine shown in Fig. 3, as prepared gravimetrically were (61.6 ± 2.0) μg g^−1^, (60.8 ± 2.0) μg g^−1^ and (78.1 ± 2.0) μg g^−1^, for Valine 1, 2 and 3, respectively (expanded uncertainties, *k* = 2). There is no clear relationship between concentration of the valine solution and the magnitude of the offset between ID-LC-CO-IRMS and qNMR results which, together with the peak area tests described earlier, suggests that the bias cannot be related to oxidation efficiency. If it were, then one would expect a smaller bias for less concentrated solutions of valine.

In terms of traceability, qNMR purity results are traceable to the SI via the kilogramme realised by use of ISO 17025 calibrated balances together with an internal standard certified for stoichiometric purity by mass fraction (e.g. the NIST SRM 350b benzoic acid used in this work). Furthermore, the application of the qNMR method within LGC has been tested via successful participation within a metrological comparison (CCQM-K55.c) demonstrating comparability and compatibility of results with other metrology institutes [37]. The ID-LC-CO-IRMS results depend on a spike material that has been characterised for purity by qNMR and for isotope ratio by LC-CO-IRMS, both of which are traceable to the SI. The mass flow of the spike and the valine solution concentration are also both traceable to the SI via the use of a calibrated balance. However, the possibility of valine loss between sampling with the autosampler syringe and injection into the mobile phase precludes the ID-LC-CO-IRMS purity result from being SI-traceable. Nevertheless, the comparison to the fully traceable qNMR approach was successful which confirms the results of the ID-LC-CO-IRMS method.

The determination of the purity of high-purity valine is something relatively straightforward to be determined by qNMR; however, there may be situations where qNMR might struggle to provide a similar level of uncertainty for purity determination as for the case described here of valine. For example, determination of the purity of a compound that forms part of a homologous series, where other compounds from the homologous series are present, would be more challenging given that the NMR signals would differ by only one additional -CH_2_ group. In such cases, reliance on qNMR alone may result in larger uncertainty in the obtained purity if insufficient care is taken to ensure that the signals used for quantitation are representative of the analyte. It is, of course, also possible to use other techniques in addition to qNMR to reduce this uncertainty during purity assessment. The ID-LC-CO-IRMS approach has the advantage that chromatography can be used to separate out the main component from other compounds present during the analysis and therefore homologous series do not require a separate analysis technique. Similarly, for molecules with more complex NMR spectra than valine, it may become difficult to identify NMR signals that can be used for quantification in the presence of impurities. These more complex molecules may also become more difficult to resolve from impurities present with the limited chromatographic separations available for the ID-LC-CO-IRMS approach.

Naturally, measurement uncertainty is not the only consideration that may be important to stakeholders of purity measurements; other aspects such as cost per analysis and turnaround time should also be considered. For ID-LC-CO-IRMS, the costs involve the consumables necessary for the instrumentation (i.e. helium and carbon dioxide gases, 18.2 MΩ cm^−1^ water, sodium persulfate, phosphoric and sulphuric acids) as well as the ^13^C-labelled glucose spike. Characterisation of the spike purity and absolute carbon isotope ratio will be a cost where such analyses cannot be performed in-house. The analysis of each substance for purity will require a separate LC-CO-IRMS analysis to determine its carbon isotope ratio (ideally using carbon isotope ratio reference materials affording traceability to the SI, rather than to the Vienna Peedee Belemnite isotope delta scale), gravimetric preparation of spike and substance solutions as well as the ID-LC-CO-IRMS analysis itself. Separation chemistry for LC-CO-IRMS systems is limited to carbon-free mobile phases and therefore, each analytical separation can require considerable time (e.g. the method suitable for the separation of amino acids described here requires 3 h). This severely limits the number of samples that can be analysed in 1 day, particularly when replicate analyses of samples and/or quality control materials are considered. For other analytes such as sugars, separations amenable to LC-CO-IRMS can be much shorter [38].

The major running cost for qNMR analyses is the cryogenic gases necessary to keep the magnet cool (liquid helium and nitrogen). The instrumentation necessary for qNMR analysis is significantly more expensive as an upfront cost than for ID-LC-CO-IRMS. The time for each analysis qNMR will be shorter—each acquisition is of the order of minutes rather than the hours needed for the LC separations described.

Both approaches will require some method development followed by verification for new substances—for the ID-LC-CO-IRMS approach, it will be the chromatographic separation of the components of interest using carbon-free, aqueous mobile phases that require the most development. For qNMR, the selection of internal standard and the signal(s) to use for quantitation will need to be investigated, particularly for larger molecules with more complex spectra.

### Proposed further improvements for ID-LC-CO-IRMS

A significant improvement to the ID-LC-CO-IRMS approach described herein would be the addition of a splitter after the LC column that diverts some of the eluent to an organic mass spectrometer to the system to allow identification of eluting compounds prior to chemical oxidation [21]. LC-CO-IRMS instrumentation without such an eluent splitter only provides isotope ratio data for peaks within the chromatogram; it cannot determine whether the peaks are associated to only one compound, or to which compound the peaks of carbon dioxide relate. The two pieces of critical information that such an additional mass spectrometer (or indeed other means of compound identification) could provide are (i) confirmation that peaks are from only one compound and (ii) the percentage carbon content of each peak.

It would then be possible to quantify any impurities detected and determine purity of an organic material by mass balance rather than the direct approach described herein of quantification of the main component. The advantage of the mass balance approach is that the large relative uncertainties of the ID-LC-CO-IRMS method would be associated with the minor impurity components, and therefore, the uncertainty in the purity of the major component would be improved by an order of magnitude. The use of a mass balance approach does have a limitation (which would equally apply to qNMR as to ID-LC-CO-IRMS) and that is the presence of impurities that may go undetected. Suitable complementary techniques to estimate the level of these impurities will therefore be needed.

A further avenue of future research is the use of online combustion interfaces for LC-IRMS instrumentation (LC-C-IRMS) which have been recently described [39–41]. This may allow the use of a wider range of analyte (and indeed spike) compounds for ID-LC-C-IRMS analyses in comparison to ID-LC-CO-IRMS as the combustion process will be more suited to some compound classes that are difficult to oxidise chemically with persulfate chemistry. Furthermore, the combustion interfaces also show promise for nitrogen isotope ratio analyses and therefore, for N-containing compounds, it may be possible to carry out quantification using a spike labelled with ^15^N, rather than (or in addition to) ^13^C. If only N-containing compounds are detected, chromatographic resolution of the compounds of interest should be easier to achieve; however, there will be larger numbers of impurities that are undetected due to containing no N limiting the effectiveness of the mass balance approach.

## Conclusions

A method for determining the purity of valine using post-column isotope dilution with a ^13^C-labelled glucose spike and LC-CO-IRMS instrumentation that does not require an internal/external standard compound of known purity has been developed and a comprehensive uncertainty budget provided. This has allowed a comparison to qNMR for the determination of valine to demonstrate the utility of the ID-LC-CO-IRMS approach for simple compound. A much lower uncertainty for the purity of valine was obtained by qNMR, but this uncertainty may increase for compounds with more complex NMR spectra than valine or where impurity signals overlap significantly with those of the target molecule. Although ID-LC-CO-IRMS resulted in an uncertainty an order of magnitude worse than qNMR, this was for direct assay of the valine, a significant reduction in achievable uncertainty is expected should the mass balance approach be used. Of the two compared methods, it is likely that qNMR will nevertheless provide the lowest uncertainty and shortest turnaround times; however, the initial cost of the instrumentation is a significantly higher hurdle to negotiate.

## Electronic supplementary material

ESM 1 (PDF 5 kb)

ESM 2 (XLSM 178 kb)
