# Supplementary material for: Systematic comparison of post-column isotope dilution using LC-CO-IRMS with qNMR for amino acid purity determination
Source: Anal Bioanal Chem. 2019 Sep 12;411(27):7207–20. doi: 10.1007/s00216-019-02116-2 (PMC6838028; doi:10.1007/s00216-019-02116-2)
Supplement: Supplementary file 1 — (PDF 5 kb) [file 216_2019_2116_MOESM1_ESM.pdf]

Analytical and Bioanalytical Chemistry

Electronic Supplementary Material

Systematic comparison of post-column isotope dilution using LC-CO-IRMS with qNMR for amino acid purity determination

Philip J H Dunn, Dmitriy Malinovski, Eli Achtar, Cailean Clarkson, Heidi Goenaga-Infante

Additional files available under 10.1007/s00216-019-02116-2.
